# Factors Influencing Green Purchase Intention: Moderating Role of Green Brand Knowledge

**DOI:** 10.3390/ijerph182010762

**Published:** 2021-10-14

**Authors:** Saeed Siyal, Munawar Javed Ahmed, Riaz Ahmad, Bushra Shahzad Khan, Chunlin Xin

**Affiliations:** 1 School of Economics and Management, Beijing University of Chemical Technology, Beijing 100029, China; 2 Department of Business Administration and Information Studies, Greenwich University, Karachi 75500, Pakistan; 3 Department of Business Administration, Iqra University, Karachi 75300, Pakistan; munawar.javed@iqra.edu.pk (M.J.A.); riaz.ahmad@iqra.edu.pk (R.A.); bushra.shahzad@iqra.edu.pk (B.S.K.)

**Keywords:** green brand knowledge, green brand positioning, environmental concern, green purchase intention, Pakistan

## Abstract

The current study aims to investigate the moderating effect of green brand knowledge (GBK) on the relationship of green brand positioning (GBP), attitude towards the green brand (ATGB), environmental concern (EC) and green purchase intention (GPI) in Pakistan. For this purpose, the data was collected from the individuals who were buying organic food by using purposive sampling, using cross-sectional research design and quantitative research approach. The Partial Least Square (PLS)-Structural Equation Modeling (SEM) technique results had shown that all the direct-effect relationships, namely, GBP, ATGB, EC variables have a positive and significant relationship with the GPI. While indirect-effect relationships have shown that the relationships of ATGB, EC and GPI are significantly moderated by GBK, which indicated that the effect of GBP, and EC toward GPI would be stronger when individuals have strong knowledge about green brands. In contrast, GBK is not significantly moderating the relationship between GBP and GPI. The empirical findings of this study fill a gap in the existing body of literature regarding the effects of GPI, ATGB and EC on green brands, as well as the moderating effect of GBK. As a result, this study provides insight into the topic, which has not been thoroughly investigated in earlier studies. Therefore, we consider that understanding this moderating effect is a positive contribution to the existing body of knowledge, which could help researchers explore this relationship in the future. This study could also help the owners and managers to know about the importance of these exogenous, and moderate variables to increase their customer’s green purchase intentions.

## 1. Introduction

The United Nations (UN) have developed 17 Sustainable Development Goals (SDGs) to transform our planet. These goals are a call for action to promote economic growth and address social needs by all countries; rich, poor, and middle-income. The 13th Goal, which is based on climate change and environmental protection, is not only attracting widespread interest but also affecting every country in all the continents due to rising sea levels, changing weather patterns and increasing levels of greenhouse gas emissions. Furthermore, the rise in global temperature to 1.5 °C has wreaked havoc on national economies. As a result, countries adopted the Paris Agreement in November 2016, and many world leaders are of the view that urgent action should be taken to limit global temperature rise to below 2 °C [1].

Actually, in the past several decades, depletion of natural resources at different levels has played an important role in environmental deterioration. For instance, Sharples, et al. [2] analyzed extreme bushfires experienced by the Australian public in recent years that resulted in loss of human life, livestock, woodlands, infrastructure, scientific facilities, cultural sites and psychological stress by firefighters and the public at large. Similarly, Hayward, et al. [3] stated that reduction in leaf litter and loss of biodiversity are increasing the threat of bushfires. In the same manner, the rainforests of the Amazon are suffering badly. Aragão, et al. [4] mentioned that a shortage of rainfall is causing a significant water deficit and affecting the photosynthetic capacity of the ecosystem. Under these circumstances, fire incidence and drought-related events are increasing in number. Additionally, keeping this climatic condition in mind, some companies are making a concerted effort to handle this grave issue. Oliver and Lee [5] as well as Alfred and Adam [6] mentioned that Honda, GM and Toyota are largely adopting green technologies that reduce the impact of greenhouse gas emissions. Likewise, Hoelzlhammer [7] indicated that Tesla is providing eco-friendly products to both urban and rural communities such as; Tesla Roadster (sports car), Tesla semi (electric truck), solar roofs and Tesla Power pack to commercial units. This initiative helps to reduce energy consumption costs and electricity bills for the consumers.

Around the globe, customers are becoming more interested in buying green brands due to green brand awareness. A green brand, according to Grant [8], is a brand that has a considerable eco-advantage throughout its competitors, as well as being capable of attracting more customers that place a high value on the creation of green purchases. According to Hartmann and Ibanez [9], a green brand comprises a set of traits as well as benefits connected with less detrimental environmental effects, and the formation of a good impression among customers, enhancing their environmental consciousness. Thus, it can be said that green customers are getting momentum nowadays as they have not only environmental consciousness but also spend money to purchase green products (GP) [10]. Additionally, Soyez [11], as well as Joshi and Rahman (2015), have suggested that consumers’ green purchase behavior is determined by product functional attributes and environmental concerns. Similarly, Kumar and Ghodeswar [12] stated that green product purchase decisions are based on multiple factors, such as environmental friendliness/social appeal by companies as well as their inclination to examine green-product-associated information from customers.

It is noteworthy to mention that environmental hazard is a key issue for both developed and developing countries. The first world countries have already taken preventive/corrective measures to handle this burning issue. For instance, people are adopting healthy lifestyles and showing an inclination towards pro-environmental behavior in the UK [13]. However, a dearth of research/practice is found in developing countries [9]. Hence, timely efforts should be made in emerging economies as well to divert the attention of consumers towards green product buying behavior [14]. Based on the above discussion, the study in hand aims to fill the gap by investigating the green buying behavior of the customers evaluating the effects of green brand positioning, attitude toward green brands, and environmental concern as the key predictors of overall green purchase intentions (GPI).

Some researchers believe that in order to promote GPI, marketers should concentrate on customer preferences as well as the decision-making process [15], because environmentally concerned consumers are changing their preferences, [16,17]. Even though these consumers are growing at a rapid pace [18], Rhead, et al. [19] suggested that researchers explore consumers’ willingness to embrace sustainable activities, attitudes, and purchasing intentions for GP, in order to address this issue. According to research, one of the most significant sustainability characteristics in green marketing literature is “green brand knowledge (GBK)”, Naliwajek-Mazurek [20]. GBK is mentioned as “a green brand node in the consumers’ memory with which a variety of associations are linked to environmental commitment and environmental concerns” [21]. Basically, GBK is an accurate determinant about particular environmental behaviors; these remain predicted by consumer attitudes to specific behaviors such as GP purchase intention [22].

Moreover, the previous studies conducted on green purchase intention established that there are direct relationships among the green brand positioning, attitude towards green brand, environmental concern, and green purchase intention [22,23,24,25,26,27], but found inconsistent results. For instance, Yusiana, Widodo, Hidayat and Oktaviani [27], Sarkar, et al. [28] showed a positive relationship. While, other studies depicted no association among these variables [25,28,29,30]. These inconsistent findings show that there is a need to re-investigate these relationships [31]. In light of the recommendations of Baron and Kenny [31], the current study introduced a moderating variable, namely, green brand knowledge to get deeper insight into the relationships between green brand positioning, attitude toward green brand, environmental concern, and green purchase intention. In addition, the previous studies were mainly conducted in other regions than Pakistan [25,32,33]. The present study focuses specifically on Pakistan to understand the green purchase intention of customers.

## 2. Literature Review

### 2.1. Theory of Reasoned Action

Various approaches have been put forward to comprehend the construct of “green product purchase intention”. For instance, Yadav and Pathak [34] relied on the “Theory of Planned Behavior (TPB)” to determine consumers’ intention towards green buying. Likewise, Suki [22] focused on the consumption values of consumers to identify their environmental concerns expressed in the purchase of green products. However, there is still a need for new avenues to be explored. This study aims to consider the “Theory of Reasoned Action” proposed by [35]. It is a belief-attitude-behavioral intention model.

TRA was established by Ajzen and Fishbein [36] to describe consumer behavioral intentions. Intentions, according to Fishbein, Jaccard, Davidson, Ajzen and Loken [35], remain the single most significant determinant of human action, next to the fact that human beings are rational regarding their use of certain accessible data, which is used in a systematic manner [37]. The model has been created with the goal of forecasting intentions for conducting reasonable decisions in everyday situations, such as utilizing birth control pills. In addition, TRA discusses the influences of cognitive mechanisms [38]. TRA examines non-routine thinking behaviors, as well as considering conduct that necessitates significant evaluation [39]. To put it another way, TRA is excellent at describing psychological/cognitive procedures so that consumers may better understand their contextual decision-making [40]. The intention of individuals to participate in a certain activity is the core concept of TRA. The term “intention” relates to a person’s willingness or preparedness to participate in the conduct in question [40,41]. According to this idea, customers’ willingness/readiness to purchase GP or embrace green decisions is measured by their GP purchase intention.

The intention is seen to be the main determinant of behavior because it precedes it [42]. TRA was already extensively researched in the field of social psychology [43,44]. Fishbein & Ajzen’s theory has been assessed in a variety of scenarios, involving health behaviors, online platforms, voting, organic food, as well as alcohol usage, among others [45,46]. Because of its high predictability, TRA has proven to be quite beneficial in predicting behavioral intents as well as behaviors within marketing and also consumer behavior [47,48]. TRA was used to predict intentions within green marketing domains such as energy-saving, recycling practices [49], as well as behaviors of green buying [50,51,52]. Nonetheless, TRA only covers volitional control and ignores ownership of necessary resources that are available [53]. The application of TRA was questioned due to the lack of key non-volitional methods of measuring human behaviors (e.g., GKB) [40,54]. Certain consumers, for example, may favor GP yet be unable to acquire them. The reason is that there is a lack of familiarity with green brands as well as products. When the simple formulation of an intention fails to predict customer behavior successfully, a moderating component adds information about the restrictions observed by consumers, enhancing the current theories prediction [55]. To broaden the scope of TRA, non-volitional variables such as GBK were added into TPB [56].

### 2.2. Green Brand Positioning and Green Purchase Intention

Green brand positioning (GBP) is concerned with the value of environmentally friendly products or services, and thus is focused on the brand’s environmentally friendly features that are useful for consumers [22,57]. Furthermore, Saha and Darnton [58] discussed the GBP as “a company’s green positioning, which represents their green image as perceived by the public.” This is defined in place of a subsection of quality, profits, as well as environmental values, which are influence green customers’ reliance on GP [57]. Lane Keller [59] stated the activities that a corporation uses to select a location in the minds of customers by examining information to develop the ideal brand awareness picture, while providing a strong reason why they must purchase a given brand, are referred to as brand positioning. GBP examines how a brand’s communication, as well as attributes, differ from those of its competitors as a result of its usage of environmentally friendly factors [22].

Consumer expectations for brand positioning should be fulfilled, according to researchers, so that consumers may identify the brand with its desirable features [26]. “The purpose of the positioning is to generate a competitive advantage in the minds of consumers over other competitor brands based on tangible or intangible product attributes,” according to popular belief [60]. Due to its green qualities and successful GBP, consumers with certain environmental knowledge as well as positive previous experiences with environmental product purchases are more likely to express strong intentions to acquire a GP [61,62]. This proclivity changes depending on the consumer’s environmental awareness and product consumption. A considerable impact on GP purchase intention has been shown in various studies [63]. Rios, et al. [64], Aulina and Yuliati [25], Hartmann and Ibanez [9] indicated that GBP reflects environmental friendly characteristics of a brand that remain highly significant to consumers. Moreover, the authors mentioned that image building through brand positioning is as important as quality and profits. Saha and Darnton [58] opined that GBP creates a positive image in the eye of the public. According to Wang [65], the consumer shall build an association with green commodities only when it is positioned according to their expectations. Gwin and Gwin [60] pointed out that GBP also means to gain a competitive advantage. Mohd Suki [62] as well as Lin and Chang [66] recommended that successful brand positioning results in consumers’ strong intention to purchase, which is caused due to environmental knowledge and positive past experiences. Mostafa [63] and Huang, Yang and Wang [23] argued that GBP shows strong correlation with GP purchase intention. Based on our review of literature, the following is hypothesized:

**Hypothesis** **1** **(H_1_).** *Green brand positioning has a significant impact on green product purchase intention*.

### 2.3. Attitude towards Green Brands and Green Purchase Intention

Kim, et al. [67] described ATGB as a term derived from customers’ appraisal and logical judgement of the GP. Consumers will be able to choose among brand alternatives due to companies’ attempts to transmit environmentally friendly aspects towards them [64]. The consumers’ preference as well as an overall assessment of the brand, which encapsulates their likes or dislikes, is linked to their attitude toward it [68]. Prior green market analysis has shown that consumers’ attitudes toward environmentally friendly behavior have a significant impact on their environmental knowledge as well as GP purchase intent [69,70]. This finding is similar to that of Yadav and Pathak [69], who found that a consumer’s attitude toward GP has a significant impact on his or her green purchasing intention. In line with these findings, Paul, et al. [71] found that Indian consumers’ attitudes strongly predict their intention to buy a green product. Certainly, according to the findings of Mostafa [63], consumers with positive views about GP are more likely to acquire a stronger tendency to purchase GP by referring to their green brands. Rezai, et al. [72] found that consumers who have a good attitude toward a brand have a higher purchase intention for that brand.

According to certain recent studies, consumers’ attitudes regarding green brands have a significant impact on their decision to purchase GP. For instance, Solomon [73] defined attitude towards brands as consumers’ likes/dislikes/preferences and overall evaluation of a brand. Honkanen and Young [74] identified that attitude influence the purchasing intention for sustainable seafood items. In the same manner, Gupta and Ogden [75], as well as Ford, et al. [76] highlighted that consumers’ attitude towards the environment affects purchasing decisions for green brands. Suki [22] mentioned that sentiments and emotional appeal craft the attitude of a consumer, thereby, impacting the intention to purchase green brands. Barber, Taylor and Strick [70] and Asih, et al. [77] also examined that attitude determines environmental knowledge and GP purchase intentions. In recent years, Paul, Modi and Patel [71] researched Indian customers wherein attitude significantly predicts GP purchase intentions, others have also investigated it [78,79]. Mostafa [63] analyzed whether customers with a positive attitude remain more receptive to purchase GP. Rezai, Kit Teng, Mohamed and Shamsudin [72] drew attention towards a positive attitude for a particular brand that subsequently effects strong purchase intention. Considering the literature, the following is postulated:

**Hypothesis** **2** **(H_2_).** *Consumers’ attitude towards green brands has a significant impact on green product purchase intention.*

### 2.4. Environmental Concern and Green Purchase Intention

An individual’s concern towards the environment as well as environmental problems is termed environmental concern (EC) [80]. In response to rising customer awareness about environmental problems as argued by [81,82,83,84], different firms have advertised themselves as environmentally friendly by marketing their GP and services. Individuals’ EC can appear in a variety of ways, ranging from specific beliefs to real behaviors (such as recycling, consuming eco-friendly products); however, the impact on consumers’ EC has already been studied, and results, in terms of customer behavior, have indeed been mixed. In a study of U.S. undergraduates, Kim and Choi [80] discovered a significant as well as direct link between EC and GP behavior. When compared to perceived consumer efficiency and collectivism, EC was the main indicator of GP behavior. Hanson [85] found that EC had a direct impact on recycling as well as GP purchases across Canadian customers. Furthermore, Maichum, et al. [86] discovered a link between EC and GP purchasing intention among Thai customers.

Other researchers, conversely, found that EC had no or only minor impacts [87]. Joubert and Schultz [88] examined nine research areas that looked at the association between EC and recycling behavior, but discovered that just five of these revealed a positive link between the two. When tested with other variables (i.e., consumer’s experience and understanding, subjective rules, attitudes forward into green purchases, perceived marketplace effect, and environmental knowledge), Joshi and Rahman [89] found that EC would be the least efficient variable in determining GP behavior between young Indian customers. Bamberg [90] reported that EC seemed to have no impact on purchase intention when using information from the green electricity brochure, and specific requests for this brochure, in a study among university students of Germany. EC, on the other hand, was linked with behavioral beliefs. According to Bamberg [90], EC influences behavior indirectly through actual problem beliefs. Others have also investigated environmental studies such as [91,92]. Alwitt and Pitts [93] argued that EC would be too broad to predict particular behaviors, so the additional variable linking EC to environmental consumption was required. They found that general EC was associated with a particular attitude to specific conduct; however, this specific attitude impacted purchase intent. Hence, the hypothesis is proposed as:

**Hypothesis** **3** **(H_3_).** *Environmental Concern has a significant impact on green product purchase intention*.

### 2.5. Moderating Effect of Green Brand Knowledge

Brand knowledge, according to Okada and Mais [94] and Gan, et al. [95], seems to be the last link between directly and indirectly utilizing a brand, such that customers remember the brand’s identity (such as symbol, color as well as the name). GBK is a concept of giving information that alters consumer behavior to be more informed about an environmentally friendly product [25]. GBK is divided into two aspects by [96], first, the green brand recognition as well as product green attributes. Green marketing could be used by a firm with an ecologically friendly brand, inside its portfolio to increase brand recognition [97]. The corporation can build awareness and provide information regarding the brand’s environmental issues [98]. Second, green brand image is described as “a collection of customer views about a particular brand that is tied to the brand’s environmental commitment” [99].

Brand image establishes a position in the minds of customers as well as obtains a competitive edge over competitors. In this way, green brands require communication and distinguishing characteristics that highlight EC Rios, Martinez, Moreno and Soriano [64]. Green brand communication activities should lead to brand knowledge and consumer engagement [100]. The focus of a green positioning strategy should be on delivering information to consumers for environmental good as well as brand qualities, and as a result, they can understand the brand’s relationship towards EC [64]. Furthermore, marketing communications create a brand image [25]. Marketing communications that frame a favorable impression in customers’ minds could be used to implement a strategy of green brand positioning. This positive awareness will result in better GBK with the help of brand awareness as well as image. Davari and Strutton [101] further explained green brand knowledge as the memory inside the mind of customers with association associated with environmental protection as well as support. Furthermore, it provides insights into the brand’s eco-friendly attributes and impacts on climate change.

These findings bear a close resemblance to Paul, Modi and Patel [71], Thongplew, et al. [102], Zhang, et al. [103], Lin and Huang [104] and the authors opined that more brand knowledge results in high green product purchase intentions. Eze and Ndubisi [105], Yadav and Pathak [69], Mohd Suki [106] further added to this notion by showing consumers’ inclination towards actual buying. Laroche, et al. [107] remarked that a positive attitude is the antecedent of purchase intention. The findings were later refined by Smith and Paladino [108] who conducted a study on the organic food industry and inferred that knowledge helps to develop attitude. On the contrary, a study by Joshi and Rahman [109] failed to recognize the association between GBK and GP purchase intention. Another scholar of the same ideology, Joshi and Rahman, 2014 [14] said that deficiency of sufficient information largely influences green purchase behavior. Likewise, Chan, et al. [110] pointed out that knowledge is insignificantly related to behavioral intention. Nonetheless, according to Huang, Yang and Wang [23], the use of brand knowledge is very plausible, as they study the moderating influence of GBK among pro-environmental attitudes, as well as strong intention to indulge in green buying behavior. Considering previous literature, the following is hypothesized:

**Hypothesis** **4** **(H_4_).** *Green brand knowledge has a significant moderating effect on the relationship between green brand positioning and green purchase intention*.

**Hypothesis** **5** **(H_5_).** *Green brand knowledge has a significant moderating effect on the relationship of attitude towards green brands and green purchase intention*.

**Hypothesis** **6** **(H_6_).** *Green brand knowledge has a significant moderating effect on the relationship of environmental concern and green purchase intention*.

**Figure 1:**
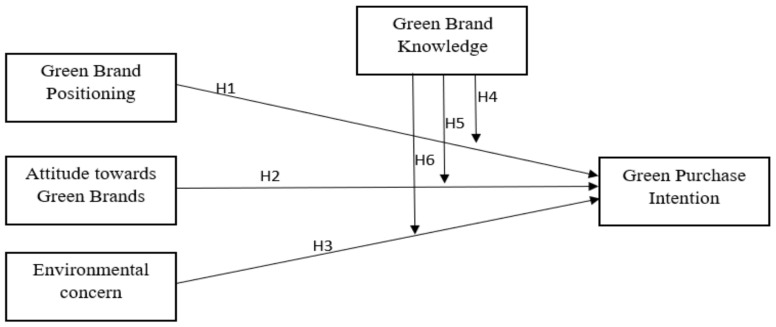
Conceptual Model.

Keeping in view the aforementioned literature and hypotheses, the following conceptual framework is proposed in this study (Figure 1).

### 2.6. Research Methodology

There are two approaches in research quantitative and qualitative. Becker, et al. [111] found that the quantitative approach places emphasis on quantifying the study and uses quantitative analysis, i.e., analyzing data with a deductive approach [112]. Based on previous approaches, the current research used a quantitative approach because this approach is considered to be better when testing the hypothesis, based on the previous theory. In other words, Ho, et al. [113] found three types of studies, exploratory, explanatory and descriptive. This paper’s aim was to investigate the moderating effect of green brand knowledge (GBK) on the relationship of green brand positioning (GBP), attitude towards green brand (ATGB), environmental concern (EC) and green purchase intention (GPI). Therefore, this study is explanatory in nature and uses PLS SEM [114,115] following [116,117]. In other words, Sekaran and Bougie [118] further shared two types of studies, cross-sectional and longitudinal. Cross-sectional study is conducted when data is to be collected once to get the responses irrespective of the time-period; it might take days, weeks, or months, but it occurs in one shot. Conversely, longitudinal study desires to study phenomena of “before/after”, therefore, data is collected more than one time. It takes a longer time-period to justify changes in behavior. Among of these two time-horizons, the current study applied the cross-sectional time-horizon by distributing questionnaires collected at one time.

The population of this study was the individuals who buy organic food at least once in a month. The non-probability (mall-intercept) technique was used to collect the data from the respondents by intercepting the respondents in the grocery stores and hypermarkets in the top three metropolitan cities of Pakistan, i.e., Islamabad, Lahore, and Karachi. A total of 500 questionnaires were distributed among the respondents by employing the mall intercept technique. A self-administered questionnaire survey was used to collect the data from the respondents. From the 500 questionnaires, 437 were completed by the respondents; however, during the final screening of the questionnaires, 41 were excluded due to the incomplete and faulty responses; finally, 396 questionnaires were selected for the statistical analysis. In the marketing studies, this number is considered sufficient for the analysis of the study as suggested by previous researchers, e.g., [118].

### 2.7. Questionnaire Development

The questionnaire developed for this study consisted of two sections. The first section of the questionnaire contained the questions to access the demographic profile of the respondents. The second section of the questionnaire consisted of the questions regarding the independent and dependent variables of this study to access the perceptions of the respondents with regards to the proposed model of this study, by asking the 23 questions. The measurement of these items was adapted from the following sources: including five items of green brand positioning, five items of attitude toward green brands, and five items of green brand knowledge adopted from [22], five items of environmental concern adopted from [17,71], and three items of green product purchase intention adopted from [25,89] (presented in Appendix A). These items were designed on a five-point Likert scale, with 1 indicating “strongly disagree” to 5 “strongly agree”. The questionnaire of this study was initially developed in English, then it was translated into Urdu language for the convenience of the respondent. The translated version was also validated by two academic professors to validate the semantic equality of the questionnaire, as suggested by [119]. After the examination by the professors, a few necessary changes were incorporated before the distribution of the questionnaires.

## 3. Data Analysis and Results

In previous research, the data had been analyzed by using different software, such as SPSS, AMOS, and Smart PLS. Among this software, we used the SPSS and Smart PLS. The SPSS was used for descriptive analysis, data screening and identifying missing data to remove the outliers, while Smart PLS was used for the inferential analysis.

### 3.1. Demographic Statistics

Descriptive analysis was performed to obtain information regarding the respondents’ demographic profiles. From the gender perspective, out of 396 respondents, 41.9 % (*n* = 166) were male and 58.1% (*n* = 230) were female. There was a big difference in age of respondents, whereby more than three-quarters of the respondents were above the age of 25. For instance, 34.1% (*n* = 135) respondents were in the age bracket of 26–30 and 33.8% (*n* = 134) were the age bracket of 31–40, only 9.3% (*n* = 370 respondents were in the age bracket of 20–25, and the remaining 22.7% *n* = 90) of respondents were in the age bracket of the 40 and above. The marital status of most of the respondents was married, represented as 68.7% (*n* = 272), and 31.3% (*n* = 124) were single. Most of the respondents were well educated, with 43.4% (*n* = 172) having a master’s degree, 33.1% (*n* = 131) having a bachelor’s degree, 14.9% (*n* = 59) having a diploma and the remaining 8.6% (*n* = 34) having other educational certificates. Regarding income, most of the respondents had a monthly income between 26,000–35,000, represented as 41.7% (*n* = 165), and the second-largest group of the respondents have an income in the range from 15,000–25,000, represented as 26.0% (*n* = 103), 20.2% (*n* = 80) respondents had an income in the range from 36,000–45,000, and the remaining 12.1% (*n* = 48) respondents earned more than 46,000 and above per month. Most importantly, the monthly green product shopping frequency was also observed in the demographic section, which reported that most of the respondents have the buying frequency of 5–7 times per month as reported 38.6% (*n* = 153) to buy the green products, 29.8% (*n* = 118) of respondents have the 3–5 time shopping frequency, 24.2% (*n* = 96) of respondents buy green products 7–10 times in a month and the remaining 7.3% (*n* = 29) are the less frequent customer of green products as they buy only 1–2 time in a month. The details of all demographic statistics are presented in Table 1 below.

### 3.2. Measurement Model

The inferential analysis of the study had been tested using a Partial Least Square (PLS)-Structural Equation Modeling (SEM). Correspondingly, Hair Jr., et al. [120]) stated that “SEM is most appropriate when the research has multiple constructs, each represented by several measured variables and allows for all of the relationship/equations to be estimated simultaneously ”. A bootstrapping analysis of 500 sub-samples was used for estimation. There are various other studies that had also used the PLS-SEM for their analysis [121]. The two-model analysis was run for conducting an inferential analysis, measurement model and structural model. The measurement model of the study could be assessed through the convergent and discriminant validity. The convergent validity could be assessed through the factor ladings that values should be greater be 0.5, average variance extracted (AVE) that value should be greater than 0.5, Cronbach alpha that value should be greater than 0.7 and composite reliability that value should be greater than 0.7. These values are recommended by various researchers in previous studies [122,123]. The Table 2 predicted values show that all the values fulfill the criteria of recommended values.

### 3.3. Discriminant Validity

For the assessment of the discriminant validity, Cross-Loadings, Fornell and Larcker [124] criterion and the Heterotrait-Monotrait ratio of correlations (HTMT) were used. Firstly, the assessment was based on cross-loadings of the items. As a rule of thumb [125,126,127], the ideal standardized loading estimates is 0.7 or higher [119]. Table 3 presents the values of the outer loadings of the items that are well above the stringent cutoff point of 0.7. However, the outer loadings exceeded 0.7 to reach the highest value of 0.910. These values were greater than the cross-loadings of other constructs, as well as complying with the rule of thumb [128]. All the loaded indicators, on their respective constructs, suggest that no cross-loadings exist among the indicators. The detail of cross-loadings is presented in Table 3.

Afterwards, the Fornell and Larcker [124] criterion was used. It suggests that a latent construct shares more variance with its indicators, rather than any other latent construct in a structural model [116]. Applying this criterion, the values of the square root of the AVE measured must be greater than the correlation of each of the construct [129]. Table 3 exhibits the discriminant validity for all constructs (i.e., values in the off-diagonal). All the squared roots of the AVE values are greater than the correlation values of the other latent variables.

Largely, the theorized measurement model for the first order constructs met the quality measures of discriminant validity. However, to remain allied with the recent literature and new techniques of assuring the quality of the measurement constructs, this study also adopted Heterotrait-Monotrait Ratio of Correlations (HTMT). It is the estimation tool to assess the factors correlation [130,131]. The HTMT is a newly developed method for the PLS-SEM to assess discriminant validity, which is one of the key building blocks of model validation. Although a cutoff of 0.90 is considered as a threshold [132] in HTMT criterion, a value of less than 0.85 is considered a careful measurement for discriminant validity [133,134,135]. The values for HTMT and corresponding confidence intervals were derived to evaluate the HTMT for all the constructs. Refer to Table 4, representing the results for all the constructs, wherein the inter-construct ratio’ values were below 0.85 and the confidence intervals contain no value of 1.0 [136]. It implies that all measuring constructs attained discriminant validity, hence, conforming to prescribed HTMT ratio of model validation.

### 3.4. Common Method Variance, Variance Inflation Factor, R Square and F Square

Harman’s Single Factor Test was also operated on Statistical Package for the Social Sciences (SPSS) to see whether the common method variance (CMV) is found or not. According to [137,138], Harman’s One-factor analysis assumes that the presence of CMB is specified by the appearance of a single-factor accounting for the widely held covariance amongst measures. With respect to this, all items in the study were tested using unrotated exploratory factor analysis employing the Principle Component Analysis technique. The researcher used SPSS to perform Harman’s single factor test. The analysis showed that the first factor contributed only 31.472% of the variance in the data that is less than 50%. Table 5 shows the value of Harman’s single factor test which is less than the recommended cumulative value of 50% resulting biasness is not found in the data and there is no CMV problem in this present paper. No single factor emerged, and the first factor did not explain most of the variance. Moreover, according to Kock [137], if the value of Variance inflation factor (VIF) that is resulting from the full collinearity test is to be less than 3.3 or equal to 3.3, then the model could be considered reliable for further analysis. The VIF values of the model are less than 3.3 (see Table 6). Hence, it is concluded that CMB was not a threat in this study. The result of one-factor analysis for CMB is depicted in Table 5.

The value of Variance inflation factor (VIF) is shown in Table 6 and it is seen that figures of all variables are clearly less than the recommended threshold value 5 [129] and 10 [130] indicating the problem of multicollinearity is not observed and the variables are not correlated.

For estimating the Structural Model R^2^ value of endogenous latent variables, figures are also examined. Table 7 shows the value of R^2^ is 50%. It means that the model explains 50% vulnerability of the feedback figures or data encompassing its mean. Consequently, we conclude that R-squared shows well the regression model appropriate to statistical details or observations because it had exceeded the minimum level, i.e., 10%, which was recommended by [131], and which had been showing the signifying explanatory power for the current model. These findings had shown the 50 percent total variance in the green purchase intention.

Different values of *f*^×^ indicate different effect sizes. Cohen (2013) explained that the values of *f*^2^ within the limits of 0.35, 0.15 and 0.02 are considered as large, medium, and small, respectively. The value of effect size (*f*^2^) is shown in Table 8, and it is seen that the values of *f*^2^ of green buying positioning (GBP) is 0.345, attitude towards green brand (ATGB) is 0.049, environmental concern (EC) is 0.021, which concludes that GBP had a large effect, ATGB and EC have a small effect. This means that the estimated model fitted the data very well.

### 3.5. Structural Model

After the assessment of the measurement model, the next step was to test the proposed hypothesis by using a structural model. For this purpose, bootstrap 10,000 resampling technique was applied for assessing the effect of independent variables “(i.e., green brand positioning, environmental concern, and attitude toward green product purchase intention)” on the dependent variable i.e., green product purchase intention), which is developed by [139]. A bootstrapping assesses the statistical significance effect of the path coefficients, and its plus or minus beta sign reveals accurate results belonging to the relationship among variables. On the other hand, the bootstrap test had been applied to determine the standard error estimation to examine the path coefficient significance from the T-test means [140,141,142]. Precisely, the path coefficient and T-values were listed as predicted in the following Table 9. The Table 9 predicted values had shown that green brand positioning (GBP) has a significant and positive (β = 0.323, *t*-value = 6.492, *p*-value = 0.000) relationship along with the green product purchase intention (GPI). Hence, H_1_ is therefore supported. In a similar vein, consumers’ attitude toward green brands (ATGB) had a significant and positive (β = 0.346, *t*-value = 6.612, *p*-value = 0.000) influence on green product purchase intention (GPI) inferring that H_2_ is also retained. Further, examination of the path coefficient shows that environmental concern (EC) is also significant and positively (β = 0.223, *t*-value = 3.425, *p*-value = 0.001) associated with the GPI that is being support to the H_3_. All of the following above discussed values are predicted in the following Table 9 below.

A test of moderation, as pointed out by [143], was undertaken to know how the moderating variable affects the relationship between endogenous and exogenous variables, in terms of strength and/or direction of the relationship. When an inconclusive relationship or weak relationship exists between exogenous and endogenous constructs, a moderator variable is typically introduced [144]. In this study, the researcher applied the moderating variable as an additional construct using the cross product of the indicator of the predictor variable and the moderator [145]. This method of testing is called a product indicator approach.

Subsequently, an interaction model was tested by creating an interaction term between green brand positioning, environmental concern, attitude toward green product and green product purchase intention. This model included the moderating effect of green brand knowledge on the relationship between green brand positioning, environmental concern, attitude toward green product and green product purchase intention and three hypotheses for moderating effect H4, H5 and H6 were tested for the moderation analysis.

**Figure 2:**
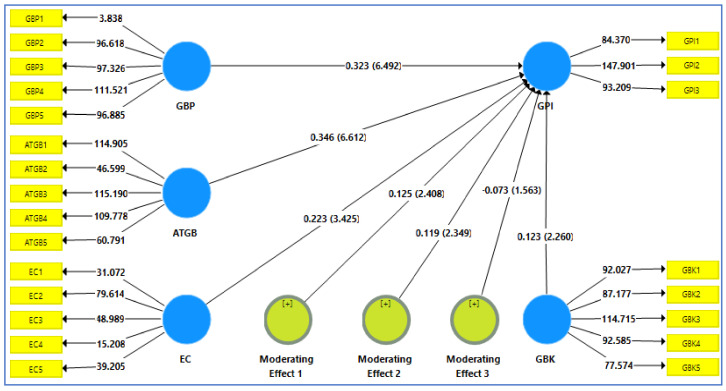
Moderating effect Model.

Based on analysis of the moderation effect, results revealed that two hypotheses (H5, and H6) of indirect relationship (moderating relationship) was accepted out of three. For instance, the result of the H5 suggests that the relationship between attitude toward green products and green product purchase intention would be strengthened by green brand knowledge as (β = 0.125, t = 2.408, *p* = 0.016) inferring that H5 is accepted. Figure 2 and Table 10 shows the green brand knowledge and attitude toward green product plot [135], where the line tagged high green brand knowledge had a steeper gradient against low green brand knowledge. This result signifies that positive nexus between attitude toward green product and green product purchase intention was stronger for customers with high green brand knowledge. In the same vein, the moderating results of the H6 revealed that the relationship between environmental concern and green product purchase intention would be strengthened by green brand knowledge as (β = 0.119, t = 2.349, *p* = 0.019) concluding that H6 is accepted. Figure 2 and Table 10 shows the green brand knowledge and environmental concern plot [136], where the line tagged high green brand knowledge had a steeper gradient against low green brand knowledge. This result signifies that the positive nexus between environmental concern and green product purchase intention was stronger for customers with high green brand knowledge. In addition, the result of the H4 suggests that the relationship between green brand positioning and green product purchase intention was not moderated by green brand knowledge as (β = −0.073, t = 1.563, *p* = 0.119) inferring that H4 is not accepted. These findings show that green brand knowledge is not considered to be a significant moderator of the relationship between green brand positioning and green purchase intention (GPI). A possible reason for this relationship is that there could be another variable overlapping in the structural model.

In this study, the Standardized Root Mean Square Residual (SRMR), the Squared Euclidean Distance (d-ULS), and the Geodesic Distance (d-G) were used to determine the model fit (NFI). The results revealed that the proposed structural model suited the data well, with acceptable indices such as SRMR = 0.052, d-ULS = 0.738, d-G = 0.665, and NFI = 0.903 [133]. As it can be seen, the SRMR value was less than the 0.08 threshold [134], the NFI value was greater than the proposed value of 0.8 [130], and the values of the CI--confidence intervals of 95% bootstrapped larger than the original values of d G and d ULS [125], indicating that the structural model satisfied the criteria.

## 4. Discussion and Conclusions

With increasing environmental concerns, consumers have become more curious about their products and services. They do prefer the products and services that are environmentally friendly. Considering growing attention towards environmental issues, the present study attempted to examine the determinants of green purchase intentions. The data were collected from the customers of the grocery stores and hypermarkets situated in the three metropolitan cities of Pakistan, namely, Lahore, Karachi, and Islamabad.

The study hypothesized a relationship between green brand positioning and green purchase intentions. The study results revealed that higher green brand positioning results in higher green purchase intentions. Thus, it supported hypothesis H1. Based on the study findings, it can be asserted that when a brand positions itself as an environment-oriented brand offering products and services to customers while addressing environmental concerns, it will tend to increase the green purchase intent among its customers. According to Liao, et al. [135], extensive emphasis on green brand positioning results in higher levels of intention among the customers to buy the green products. Similarly, Huang, Yang and Wang [23], Aulina and Yuliati [25] and Himawan [136] in their studies reported that brand green positioning leads to green purchase intentions. Hence, the present study findings are supported by previous studies [136].

Additionally, the study hypothesized that attitudes towards both the green brand and environmental concerns positively contributes towards green purchase intentions. The results of the study posit that environmental concerns and attitudes towards green brands determine the green purchase intentions. The study findings are supported by previous studies. For instance, Himawan [136] put forward that attitude towards green brands predicts green purchase intentions. Moreover, Aulina and Yuliati [25] reported that when consumers do have higher levels of attitude towards green brands, they tend to develop an intent to buy green products. It is asserted that when consumers have a positive attitude towards the brand, it results in green purchase intentions. Their positive green attitude towards the brand due to environmental concerns tend to generate green purchase intentions [137]. Recently, it was argued that attitude is regarded as the exact part that plays a key role in performing a specific behavior and consumers who possess the higher positive environmental attitude tend to have higher green purchase intentions. On the other hand, the study also hypothesized about the relationship between environmental concerns and green purchase intentions. The study findings posited that higher environmental concerns are related to increased green purchase intentions. These findings are supported by the previous studies, for instance, Lee and Lim [138] reported that environmental concerns indirectly influence consumers purchasing behaviors. On the other hand, Liao, Wu and Pham [146] contended that higher environmental concerns among customers facilitate their development towards green purchase intentions. Hence, hypotheses H2 and H3 are supported and accepted.

Besides measuring the direct influence between attitude towards the green brand, environmental concerns and green brand positioning, the study has also examined a moderating influence of green brand knowledge. The study hypothesized that green brand knowledge strengthens the relationship between independent variables (ATGB, EC and GBP) and green purchase intentions. Accordingly, the study results also presented a positive significant moderation between independents (ATGB, and EC) and green purchase intentions. These findings suggest that when the customers do have the knowledge about the products and services, the relationship between their attitude and intention will become stronger. Notably, consistent provision of the information about the green brands ultimately becomes a piece of knowledge for them. Consumer’s knowledge regarding the environment moderates attitudes and green behaviors [139]. Accordingly, the study also put forward that the relationship between environmental concerns and green purchase intentions is moderated by the green brand knowledge. It is consistent with previous studies, however, the study did not present the green brand knowledge as a moderator between the relationship of GBP and GPI. It means that the green brand positioning influences green purchase intentions, but it does not vary significantly with an increase in environmental knowledge. These findings are consistent with the previous study [22,147].

### 4.1. Theoretical and Practical Implications

The study has various theoretical and practical implications. First, the study has presented empirical evidence on green purchase intentions from the theory of reasoned actions. It is a valuable contribution to the theory of reasoned action. It enhanced the empirical literature and understanding of how different factors can result in green purchase intentions from the theory of reasoned actions. Moreover, the study has provided empirical evidence on the moderating role of green brand knowledge. It enhanced the understanding of how green brand knowledge among the individuals leads them towards green purchase intentions in the presence of brand positioning, attitude and environmental concerns. Additionally, the current study could also provide help to the researcher to conduct their future research.

From a practical perspective, the study presented evidence that green brand positioning is one of the important predictors of green purchase intentions. It is necessary that marketers ensure the green positioning of their brands as they result in employee green purchase intentions. Accordingly, Suki [22] contended that the majority of the organizations tend to directly promote their brands by using the traditional or electronic means of communication that enhance their brand positioning. It is suggested that marketers must incorporate the eco-friendliness of the products or services while positioning their brands. Hence, they will be able to ensure green positioning and better drive green purchase intentions. Additionally, Suki [22] also contended that when a brand positions itself as a brand that meets the environmental expectations of the customers, then it gets trusted by its customers. Therefore, when the brands are environmentally positioned (Green positioning), such that they are able to address the environmental issues and meet them, then the consumer intentions to buy such products and services will be higher.

Besides the green brand positioning, the study also presented the attitude towards the green brands and environmental concerns as the predictors of the green purchase intentions. The organizations are needed to increase the positive green brand attitude that will translate into green purchase intentions. The brands are required to pay special attention to building the green band attitude by using focused and integrated marketing and communication strategies. Because the green brand attitude will positively result in green purchase intentions among the consumers. They may use social media as a widely used channel of communication to enhance the knowledge of the consumers since it is found to be a catalyst that can increase the green purchase intentions. Marketers should make efforts to increase the environmental concerns and they can accomplish it by showing that the environment is facing a hazardous situation [140]. Therefore, educating consumers through the social media channels of the brand will enhance their knowledge, resulting in an intention to buy the green products and services.

### 4.2. Limitations and Future Directions

The results of the study should be interpreted by considering the limitations. First, the study used the cross-sectional research quantitative research design to test the hypothesis. It is recommended that future studies use the longitudinal research design to observe if there is any change in the respondents’ responses happen over the time of the study and it will also help to test and verify the causal relationship between variables. Additionally, the study has used PLS-SEM as a statistical tool. Although it is being widely used as a tool for statistical analysis, it is recommended that future studies use more robust tests for establishing the relationship between variables. More variables and their dimensions can be studied further. For instance, the study has considered the green brand positioning as a uni-dimensional construct, which may limit the generalization from different perspectives, since it carries dimensions such as functional, emotional and green positioning [23]. Therefore, it is recommended that future studies consider the multi-dimensional construct of green brand positioning, which will enhance the understanding of how positioning from different perspectives can drive the green purchase intentions. On theoretical grounds, it is suggested that authors look into other theories to predict the green purchase behavior of the individuals for instance, theory of planned behavior [141]. Moreover, the green brand positioning was not moderated by green brand knowledge, therefore, future research could consider other moderating or mediating variables to increase the predictive relevance of the model. Finally, the study had applied the cross-sectional research design in which data had been collected at one time. There are various other research designs, such as longitudinal, in which data could be collected more than once; in this regard, future research could be undertaken on longitudinal research design.

## Figures and Tables

**Table 1:** Demographic profile of the respondents.

Demographic Variables	Category	Frequency	Percentage
Gender	Female	230	58.1%
	Male	166	41.9%
Age	20–25	37	9.3%
	26–30	135	34.1%
	31–40	134	33.8%
	41 and above	90	22.7%
Marital status	Married	272	68.7%
	Single	124	31.3%
Education level	Diploma	59	14.9%
	Bachelors	131	33.1%
	Masters	172	43.4%
	Others	34	8.6%
Income per month	15,000–25,000	103	26.0%
	26,000–35,000	165	41.7%
	36,000–45,000	80	20.2%
	46,000 and above	48	12.1%
Green shopping frequency	1–2 times	29	7.3%
	3–5 times	118	29.8%
	5–7 times	153	38.6%
	7–10 times	96	24.2%

Source: Own Illustration.

**Table 2:** Measurement model (factor loading, Cronbach’s Alpha, CR and AVE).

Construct Name	Items	Loading	C-Alpha	CR	AVE
GBP	GBP1 GBP2 GBP3 GBP4 GBP5	0.652 0.904 0.913 0.914 0.907	0.912	0.936	0.747
ATGB	ATGB1 ATGB2 ATGB3 ATGB4 ATGB5	0.924 0.857 0.916 0.923 0.874	0.941	0.955	0.809
EC	EC1 EC2 EC3 EC4 EC5	0.825 0.883 0.86 0.675 0.821	0.875	0.908	0.666
GBK	GBK1 GBK2 GBK3 GBK4 GBK5	0.919 0.894 0.911 0.908 0.892	0.945	0.958	0.819
GPI	GPI1 GPI2 GPI3	0.895 0.940 0.905	0.901	0.938	0.835

Note: GBP—green brand positioning, ATGB—attitude towards green brand, EC—environmental concern, GBK—green brand knowledge, GPI—green purchase intention.

**Table 3:** Discriminant validity Fornell and FornellLarcker [126].

	ATGB	EC	GBK	GBP	GPI
ATGB	0.899				
EC	0.418	0.816			
GBK	0.476	0.554	0.905		
GBP	0.533	0.454	0.427	0.864	
GPI	0.583	0.487	0.477	0.618	0.914

Note: GBP—green brand positioning, ATGB—attitude towards green brand, EC—environmental concern, GBK—green brand knowledge, GPI—green purchase intention.

**Table 4:** Discriminant validity (HTMT).

	ATGB	EC	GBK	GBP	GPI
ATGB					
EC	0.442				
GBK	0.502	0.589			
GBP	0.569	0.495	0.457		
GPI	0.628	0.524	0.513	0.676	

Note: GBP—green brand positioning, ATGB—attitude towards green brand, EC—environmental concern, GBK—green brand knowledge, GPI—green purchase intention.

**Table 5:** Harman’s Single Factor Test.

Criterion	Acceptability
Harman’s Single Factor Test	31.472% variance proportion

**Table 6:** Collinearity Assessment for Inner model (Variance inflation factor (VIF) values).

VIF < 5	GPI
GBK	2.998
GBP	2.182
ATGB	1.877
EC	1.599

Note: GBP—green brand positioning, ATGB—attitude towards green brand, EC-environmental concern, GPI—green purchase intention.

**Table 7:** R^2^ and Adjusted R^2^ values.

	R Square
GPI	0.506

GPI—green purchase intention.

**Table 8:** Effect size (*f*^2^).

	GPI
GBP	0.345
ATGB	0.049
EC	0.021

Note: GBP—green brand positioning, ATGB—attitude towards green brand, EC—environmental concern, GPI—green purchase intention.

**Table 9:** Direct relationship results.

Hypotheses	Relationship	Beta	STDEV	T Statistics	*p*-Values	Decision
H1	GBP → GPI	0.323	0.05	6.492	0.000	Supported
H2	ATGB → GPI	0.346	0.052	6.612	0.000	Supported
H3	EC → GPI	0.223	0.065	3.425	0.001	Supported

Note: GBP—green brand positioning, ATGB—attitude towards green brand, EC—environmental concern, GPI—green purchase intention.

**Table 10:** Moderation results.

Hypotheses	Relationship	Beta	STDEV	T Statistics	*p*-Values	Decision
H4	GBP × GBK -> GPI	−0.073	0.047	1.563	0.119	Not Accepted
H5	ATGB × GBK -> GPI	0.125	0.052	2.408	0.016	Accepted
H6	EC × GBK -> GPI	0.119	0.051	2.349	0.019	Accepted

Note: GBP—green brand positioning, ATGB—attitude towards green brand, EC—environmental concern, GBK—green brand knowledge, GPI—green purchase intention.

## Appendix A

**Table A1:** Measurement Scales.

No.	Construct	Items
1	Green Brand Positioning	Quality and price are important considerations when I purchase green products. I get to know about green branding through advertisement. Green products have matched my personal. Wants and needs green product always overpriced. I prefer to purchase environmentally green products.
2	Attitude toward green brand	I feel that green product’s environmental reputation is generally reliable. I feel that green product’s environmental performance is generally dependable. I feel that green product’s environmental claims are generally trustworthy. Green product’s environmental concern meets my expectations. Green products keep promises and responsibilities for environmental protection.
3	Green brand knowledge	Going green products could be a beneficial investment in long-term. Green product’s environmental performance meets my expectations. Lack of availability of access is a major reason for low popularity and demand of green products. I purchase green products because they are environmentally friendly. I purchase green products because they have more environmental benefit than other products.
4	Environmental concern	I am very concerned about the environment. I would be willing to reduce my consumption to help protect the environment. Major political change is necessary to protect the natural environment. Major social changes are necessary to protect the natural environment. Anti-pollution laws should be enforced more strongly.
5	Green products purchase intention	I intend to buy green products because of my environmental concern. I expect to purchase green products in the future because of their environmental benefits. Overall, I am glad to purchase green products because they are environmentally friendly.
